# Iatrogenic salt water drowning and the hazards of a high central venous pressure

**DOI:** 10.1186/s13613-014-0021-0

**Published:** 2014-06-21

**Authors:** Paul E Marik

**Affiliations:** 1 Division of Pulmonary and Critical Care Medicine, Eastern Virginia Medical School, 825 Fairfax Av, Suite 410, Norfolk 23507, VA, USA

**Keywords:** Fluid, Fluid balance, Normal saline, Lung water, Extra-vascular lung water, Central venous pressure, ICU, Lactate Ringers Solution, Acute respiratory distress syndrome, Sepsis, Mean circulatory filling pressure, Fluid overload

## Abstract

Current teaching and guidelines suggest that aggressive fluid resuscitation is the best initial approach to the patient with hemodynamic instability. The source of this wisdom is difficult to discern, however, Early Goal Directed therapy (EGDT) as championed by Rivers *et al*. and the Surviving Sepsis Campaign Guidelines appears to have established this as the irrefutable truth. However, over the last decade it has become clear that aggressive fluid resuscitation leading to fluid overload is associated with increased morbidity and mortality across a diverse group of patients, including patients with severe sepsis as well as elective surgical and trauma patients and those with pancreatitis. Excessive fluid administration results in increased interstitial fluid in vital organs leading to impaired renal, hepatic and cardiac function. Increased extra-vascular lung water (EVLW) is particularly lethal, leading to iatrogenic salt water drowning. EGDT and the Surviving Sepsis Campaign Guidelines recommend targeting a central venous pressure (CVP) > 8 mmHg. A CVP > 8 mmHg has been demonstrated to decrease microcirculatory flow, as well as renal blood flow and is associated with an increased risk of renal failure and death. Normal saline (0.9% salt solution) as compared to balanced electrolyte solutions is associated with a greater risk of acute kidney injury and death. This paper reviews the adverse effects of large volume resuscitation, a high CVP and the excessive use of normal saline.

## 

In the critically ill and injured patient, aggressive supportive measures may be harmful and the ‘less is more’ paradigm appears applicable. In these highly vulnerable patients, more intensive treatments may promote the chances of unwanted adverse effects and hence, iatrogenic injury [1]. Traditional teaching suggests that aggressive fluid resuscitation is the best initial approach for the cardiovascular instability of sepsis. In the Rivers’ Early Goal Directed Therapy (EGDT) study, 4.9 liters of crystalloid were given in the first 6 hours and 13.4 liters in the first 72 hours [2]. The Surviving Sepsis Campaign recommends ‘aggressive fluid resuscitation during the first 24 hours of management’ [3]. The guidelines for hemodynamic support of sepsis published by the American College of Critical Care Medicine state that ‘large fluid deficits exist in patients with septic shock. Up to 6 to 10 liters of crystalloid solutions may be required for initial resuscitation in the first 24 hours’ [4]. Consequently, large volumes of fluid are often infused in the early stages of sepsis. Traditionally, patients undergoing surgery have been managed with a liberal fluid strategy in which fluids are administered to fill the non-existent ‘third space’ [5]–[7]. For patients with traumatic injuries, high volume fluid resuscitation is promoted by the early Advanced Trauma Life Support (ATLS) strategy [8]. There are, however, no human data to show that large volume fluid resuscitation reliably improves organ perfusion [9],[10]. This approach is likely to lead to iatrogenic salt water drowning with the acute respiratory distress syndrome (ARDS), acute kidney injury (AKI), myocardial injury, gastrointestinal dysfunction, poor wound healing with an increased the risk of death [6],[11]–[21]. Aggressive fluid resuscitation is a well-known risk factor for secondary intra-abdominal hypertension which in turn is associated with hepatic and respiratory dysfunction, AKI, multiorgan failure and death [22]–[27]. Current guidelines suggest targeting a central venous pressure (CVP) of 8 to 12 mmHg in patients with severe sepsis and septic shock [28]. However, a high CVP increases venous pressure, increases organ interstitial pressure and reduces organ and microcirculatory flow [29],[30]. A CVP > 8 mmHg has been associated with an increased risk of renal failure and death [13],[16],[31]. Normal saline (0.9% salt solution) is common resuscitation fluid. However, as compared to balanced electrolyte solutions normal saline (NS) is associated with a greater risk of acute kidney injury and death [32]–[35]. This paper reviews the adverse effects of large volume resuscitation, a high CVP and the excessive use of normal saline.

## Review

### The dangers of large volume resuscitation

**Figure 1:**
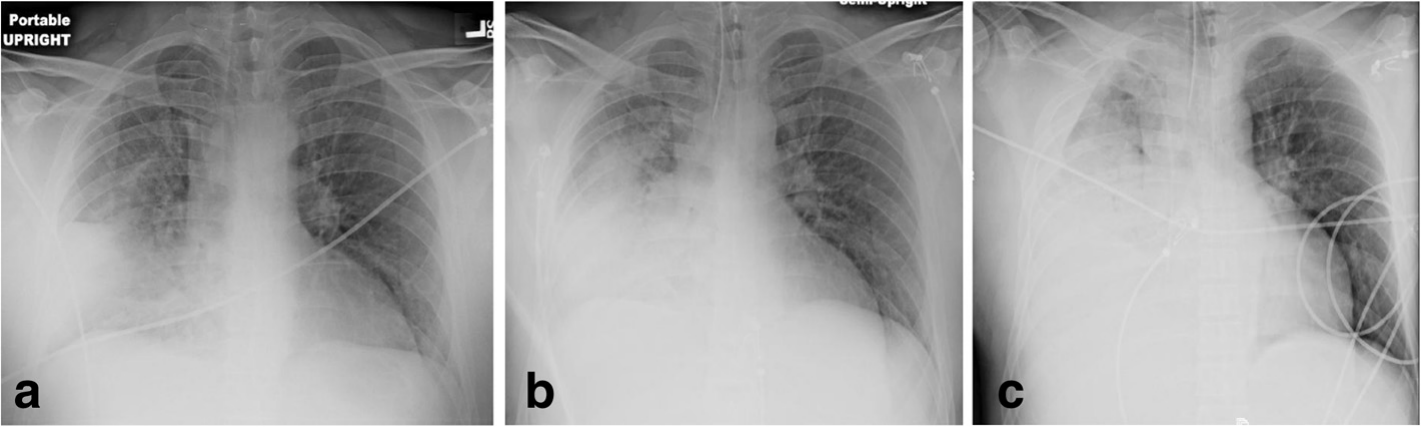
**Fourty-four-year old male with Pneumococcal pneumonia. (a)** Initial chest radiograph (CXR) in emergency department. **(b)** CXR four hours later after four liters of crystalloid (patient now intubated). **(c)** CXR 12 hours after admission, after 9 liters of crystalloid, central venous pressure (CVP) = 10 mmHg. Patient died six hours later of refractory hypoxemia.

From a pathophysiological point of view, large volume fluid resuscitation in patients with sepsis is illogical and may worsen the hemodynamic derangements of sepsis. Sepsis is primarily a vasoplegic state due to increased production of nitric oxide, activation of K_ATP_ channels and vasopressin deficiency [36]. Vasoplegic shock, due to failure of the vascular smooth muscle to constrict, results in arterial and venodilation [36]. Venodilation increases the unstressed vascular compartment, thus decreasing venous return. In patients with septic shock who are fluid responders, vasodilatation with a fall in systematic vascular resistance has been observed following fluid resuscitation [37],[38]. A similar finding has been noted in an experimental sepsis model [21]. Furthermore, it is important to emphasize that the septic heart responds poorly to fluid loading. In patients with sepsis, the Frank-Starling curve is shifted downwards and to the right, with septic patients showing a diminished response to fluid loading. This observation was demonstrated by Ognibene and colleagues over 25 years ago [39]. Patients in septic shock had a minimal increase in left ventricular stroke work index (LVSWI) in response to volume infusion. This suggests that large volume fluid resuscitation will cause a small increase in stroke volume (with further vasodilatation) at the expense of large increases in filling pressures (increase in both the CVP and left atrial pressures). Furthermore, sepsis is characterized by increased endothelial permeability caused by shedding of the endothelial glycocalyx and the development of gaps between endothelial cells (paracellular leak) [40],[41]. An increase in microcirculatory hydrostatic pressure following aggressive fluid resuscitation increases fluid extravasation. Increased cardiac filling pressures following aggressive fluid resuscitation increase the release of natriuretic peptides which act synergistically with nitric oxide causing cGMP mediated vasodilatation [36]. In addition, natriuretic peptides cleave membrane-bound proteoglycans and glycoproteins (most notably syndecan-1 and hyaluronic acid) off the endothelial glycocalyx [42]–[44]. This profoundly increases endothelial permeability. Increased natriuretic peptides inhibit the lymphatic propulsive motor activity reducing lymphatic drainage [45]–[47]. Increased natriuretic peptides following aggressive fluid resuscitation therefore acts to sequestrate fluid into the interstitium. Furthermore, increased filling pressures and a positive fluid balance increase extra-vascular lung water (EVLW) [18]. Increased EVLW impairs gas exchange, reduces lung compliance and increases the work of breathing [18]. Increased EVLW is regarded as a defining feature of acute lung injury/ARDS [48],[49]. Increased EVLW is a strong independent predictor of death [12],[18],[50],[51]. Due to the endothelial injury, capillary leak and increased hydrostatic pressures less than five percent of infused crystalloid remains intravascular within three hours after infusion resulting in further increases in EVLW and tissue edema [52]. Tissue edema impairs oxygen and metabolite diffusion, distorts tissue architecture, impedes capillary blood flow and lymphatic drainage and disturbs cell-cell interactions; these effects contribute to progressive organ dysfunction [14]. These effects are pronounced in encapsulated organs, such as the liver and kidneys, which lack the capacity to accommodate additional volume without an increase in interstitial pressure, resulting in compromised organ blood flow [14]. This may lead to AKI and hepatic congestion with cholestasis and impaired hepatic function [14]. Myocardial edema due to excess fluid administration compounds the myocardial dysfunction common in critically ill patients [21]. In a cohort of patients requiring mechanical ventilation, Cordemans and colleagues reported significantly more respiratory, liver and cardiovascular organ-failure free days in patients’ with a conservative fluid strategy and those whose EVLW fell by more than 2 ml/kg during their ICU stay [18]. Bowel edema results in malabsorption, ileus and bacterial translocation. In patients with pneumonia, large volume fluid resuscitation may result in severe ARDS (see Figure 1). The chest radiograph series presented in Figure 1 represents a typical case of fatal iatrogenic salt water drowning. This patient was resuscitated according to the EGDT and the Surviving Sepsis Campaign bundles, in which fluids are administered until the CVP > 12 mmHg [2],[28].

The harmful effects of aggressive fluid resuscitation on the outcome of sepsis are supported by experimental studies as well as data accumulated from clinical trials [15],[20],[21],[31],[51],[53]–[58]. Multiple clinical studies have demonstrated an independent association between an increasingly positive fluid balance and increased mortality in patient with sepsis [15],[18],[31],[51],[53]–[58]. In a secondary analysis of the Vasopressin in Septic Shock Trial (VASST), Boyd and colleagues demonstrated that a greater positive fluid balance at both 12 hours and 4 days were independent predictors of death [31]. Furthermore, a number of studies have demonstrated that a positive fluid balance was associated with an increased risk for AKI [15]. Bouchard *et al*. demonstrated that in patients with AKI, fluid overload was independently associated with mortality [58]. In the Fluid and Catheter Treatment Trial (FACTT), the conservative fluid strategy was associated with a trend towards a reduced requirement for renal replacement therapy [59]. The most compelling data that fluid loading in sepsis is harmful come from The Fluid Expansion as Supportive Therapy (FEAST) study performed in 3,141 sub-Saharan children with severe sepsis [60]. In this randomized study, aggressive fluid loading was associated with a significantly increased risk of death. Furthermore, there was no subgroup of patients that benefited from aggressive fluid resuscitation [61].

Emerging data suggest that less than 50% of septic patients who present to the emergency department (and are fluid naive) will be fluid responsive [62]. In some patients, hypotension and tachycardia do resolve with limited fluid resuscitation. However, fluids alone will not reverse the hemodynamic instability of patients with more severe sepsis; in these patients’ fluids alone are likely to exacerbate the vasodilatory shock and increase the capillary leak, tissue edema and organ dysfunction [21]. In the Australasian Resuscitation of Sepsis Evaluation (ARISE) study which used the same entry criteria as the EGDT study, 2.2 ± 1.9 liters of fluid were given in the first 6 hours [63]. This compares to 4.9 liters in the intervention arm of the EGDT study [2]. The hospital mortality was 23% in the ARISE study compared to 30% in the intervention arm of the EGDT study. This difference in the prescription of fluid is likely due to variability in the management style of severe sepsis between countries. In a survey conducted by Reade *et al*., intensivists from Australia tended to give less fluid than Americans with only 15% of Australians targeting a CVP of 8 to 12 mmHg [64]. In the VASST study optimal survival occurred with a positive fluid balance of approximately three liters at twelve hours [31]. Recently, the Protocolized Care for Early Septic Shock (ProCESS) study was published which compared protocol-based EGDT (Rivers’ protocol) and protocol-based standard therapy with usual care [65]. It is noteworthy that the amount of fluid given in the first 6 hours and from 7 to 72 hours was significantly less in the ProCESS EGDT patients than in the Rivers’ EGDT patients (see Table 1). As patients were resuscitated according to the same protocol this would appear to be anomalous. However, it should be noted that the mean CVP at 6 hours was 13.8 ± 4.4 mmHg in the Rivers’ EGDT group. Assuming a normal distribution, 50% of patients in the Rivers’ EGDT would have achieved CVPs greater than the mean value of 13.8 mmHg. Thus most patients in the Rivers’ EGDT study had CVPs outside the stated goal (>8 to 12 mmHg). Furthermore, the use of vasopressors in the first six hours of EGDT was significantly greater in ProCESS than in the Rivers’ study (see Table 1). We hypothesize that targeting a lower mean CVP in the EGDT arm of the ProCESS trial resulted in less fluid being given and the earlier use of vasopressors. It is therefore possible that the large amount of fluid administered in the Rivers’ study partly accounted for the mortality difference between the EGDT arms of the Rivers’ and ProCESS studies.

**Table 1:** Contrasting use of fluids and vasopressors (and mortality) in the Early Goal Directed Therapy (EGDT) arms of the Rivers’ and ProCESS studies

**Study**	**Fluid 0 to 6 hours (ml)**	**Fluid 7 to 72 hours (ml)**	**Fluid 0 to 72 hours (ml)**	**Vasopressors (%) 0 to 6 hours**	**60-day mortality (%)**
Rivers’ EGDT	4,981	8,625	13,443	27.4	44.3
ProCESS EGDT	2,805	4,428	7,220	54.9	21

Multiple RCT’s and cohort studies have demonstrated that a conservative fluid strategy in patients undergoing elective non-cardiac surgery is associated with significantly fewer complications with a lower mortality (in the high risk patients) than patients managed with the traditional liberal fluid strategy in which fluids are administered to fill the non-existent ‘third space’ [5]–[7]. For patients with traumatic injuries, high volume fluid resuscitation as promoted by the early Advanced Trauma Life Support (ATLS) strategy [8], has given way to a ‘damage control’ resuscitation strategy [66]. This approach has seen a fall in the volume of crystalloid delivered in the emergency department and an associated fall in mortality. In a prospective analysis of 3,137 trauma patients treated in the Emergency Department, fluid volumes of 1.5 liters or more were significantly associated with mortality [67]. This observation is supported by a meta-analysis which demonstrated in both RCTs and cohort studies that a conservative fluid strategy was associated with a lower mortality in trauma patients [68]. Similarly, an aggressive fluid strategy in the resuscitation of patients with acute pancreatitis has been associated with an increased risk of complications [69].

The results of multiple studies across diverse patient populations have clearly demonstrated that aggressive fluid resuscitation is associated with an increased risk of complications and death. The only published study conducted in adult patients that has demonstrated that early aggressive fluid resuscitation improves outcome is the EGDT study by Rivers *et al*. [2]. However, the results of this study do not appear to be biologically plausible (implausible effect size) [70], the elements of the protocol were not based on evidence-based interventions [71], the analysis of the results of study has come under scrutiny [72], and the results were not validated in a large randomized controlled trial [65]. While hypovolemia will result in decreased cardiac output (and blood pressure) with inadequate organ perfusion leading to organ dysfunction, overzealous fluid resuscitation and hypervolemia induces a cascade of events that similarly results in organ dysfunction. From an evolutionary point of view, humans have evolved to deal with hypovolemia and not hypervolemia. Hypervolemia is a recent (last 20 years) and largely iatrogenic phenomenon. The argument is no longer ‘wet or dry’ but ‘just the right amount of fluid’ [73],[74].

### The dangers of a high CVP

Not only has the CVP failed as a useful measure for the assessment of preload and fluid responsiveness [75], but a CVP > 8 mmHg is independently associated with a higher mortality and increased risk of AKI in patients with sepsis and heart failure [13],[31],[76]. This suggests that the CVP component of the six-hour resuscitation bundle as widely promoted by the Surviving Sepsis Campaign may lead to harm [28]. It is important to note that a normal CVP is close to zero and not 8 to 12 mmHg as the Surviving Sepsis Campaign might lead one to believe [28]. The mean circulatory filling pressure (MCFP) is regarded as the driving pressure that determines venous return and is considered synonymous with the effective circulatory blood volume [77]–[81]. The MCFP is conceptualized as the pressure distending the vasculature when the heart is stopped (zero flow) and the pressures in all segments of the circulatory system have equalized [78]–[80]. The MCFP in humans is normally in the range of 8 to 10 mmHg [80]. According to Guyton, venous return is determined by the gradient between MCFP and CVP [78]. An increase in the CVP or a fall in the MCFP will reduce venous return, stroke volume and cardiac output. Cecconi and colleagues investigated the relationship between the changes in MCFP with the change in CVP and stroke volume following a fluid challenge in postsurgical ICU patients [82]. In this study MCFP increased equally in fluid responders and non-responders (3.1 ± 1.9 versus 3.1 ± 1.8 mmHg). However, the increase in the CVP was greater in the non-responders than the responders. This study emphasizes that a disproportionate increase in the CVP will impede venous return and cardiac output [81].

In addition to influencing venous return, a high CVP is transmitted backwards increasing venous pressure. The increase in venous pressure has a profound effect on microcirculatory flow and organ function. The kidney is particularly affected by congestion and increased venous pressure, which leads to increased renal subcapsular pressure and lowered renal blood flow and glomerular filtration rate (GFR) [83]. Furthermore, increased renal interstitial pressure may collapse intrarenal collecting lymphatics which compromise lymph flow [84]. The detrimental effect of high venous pressure on renal function was established by FR Winton in an elegant set of experiments performed in 1930s [29]. This investigator attached the kidneys of a dog to a heart-lung circulation by means of cannulae inserted into the artery and veins of the kidneys and then independently altered venous and arterial pressure. Dr Winton demonstrated that increasing venous pressure dramatically decreased urine production. More recently, Legrand and colleagues investigated the association between hemodynamic variables and AKI in patients with sepsis [13]. In this study, the CVP was the only hemodynamic variable associated with the development of AKI; cardiac output, mixed venous oxygen saturation (ScvO_2_) and mean arterial pressure (MAP) were unable to predict the development of AKI. These authors noted a linear relationship between increasing CVP and AKI; there was a trend for higher CVP to be associated with worse renal outcome for all levels of CVP above 4 mmHg, with a CVP of 15 mmHg being associated with an 80% risk of new or persistent AKI, compared to approximately 30% at a CVP of 6 mmHg. Van Biesen and colleagues demonstrated that septic patients developing AKI had a higher positive fluid balance, a higher CVP and worse oxygenation than septic patients without AKI [16]. It is important to point out that these are observational data, and that it is likely that other factors play a role in the pathophysiology of AKI in sepsis. In patients with acute decompensated heart failure, Mullens *et al*. demonstrated a near linear relationship between increasing CVP and worsening renal function [76]. In this study, worsening renal function occurred significantly less frequently in patients with a CVP < 8 mmHg. Furthermore, similar to the findings of Legrand and colleagues, the CVP was the only hemodynamic parameter that predicted worsening renal failure, with the cardiac index, systolic blood pressure and pulmonary capillary wedge pressure being similar between those patients who maintained renal function as compared to those with worsening renal function. In a subanalysis of the Evaluation Study of Congestive Heart Failure and Pulmonary Artery Catheterization Effectiveness trial (ESCAPE), Nohria *et al*. demonstrated a significant correlation between baseline renal function and the CVP, there was, however no correlation between baseline renal function and cardiac index, pulmonary capillary wedge pressure or systemic vascular resistance [85]. These data suggest that a high CVP independently increases the risk for ‘congestive kidney failure’. Most clinicians fluid load patients with oliguria; this intervention sets into motion a vicious cycle, with fluid loading further increasing renal venous pressure with a further decline in renal function and urine output [16].

In addition to increasing renal venous and interstitial pressure, a high CVP will result in an increase in hepatic and intestinal venous pressure causing hepatic and intestinal congestion and impaired microcirculatory flow. Indeed, in a study of 70 patients with sepsis, Vellinga and colleagues demonstrated that the sublingual microvascular flow index (MFI) and percentage of perfused vessels (PPV) was significantly lower with a patients with a high CVP (>12 mmHg) than a low CVP: 1.44 ± 0.94 versus 1.89 ± 0.91, *P* = 0.006; and 88 ± 21 versus 95 ± 8%, *P* = 0.006) [30]. The cardiac index, MAP and perfusion pressure (MAP-CVP) did not differ significantly between the high and low CVP groups. In a multivariate logistic regression analysis, the only significant predictor for an abnormal MFI was a CVP > 12 mmHg. Because microcirculatory driving pressure is the difference between post-arteriolar and venular pressure, a relatively mild increase in CVP may considerably influence the capillary perfusion pressure and microcirculatory flow [30]. As the pressure drop in the vascular system occurs upstream at the level of small arterioles (resistance vessels), the microcirculation is considered a low pressure compartment. Therefore, mean capillary pressure is much closer to venous than to arterial pressure. Therefore, as long as the MAP is within an organ’s autoregulatory range, the CVP becomes the major determinant of capillary blood flow. This suggests that venous pressure has a much greater effect on microcirculatory flow than the MAP. The adverse consequences of volume overload and a high CVP are summarized listed below

Consequences of volume overload

 Pulmonary edema and increased extra-vascular lung water

 Impaired oxygenation

 Altered pulmonary and chest wall mechanics

 Increased work of breathing

 Myocardial edema

 Decreased contractility

 Diastolic dysfunction

 Conduction defects

 Increased intraabdominal pressure

 Acute kidney injury

 Hepatic dysfunction

 Decreased lung volumes

 Ileus

 Gastrointestinal

 Ileus

 Malabsorption

 Bacterial translocation

 Hepatic congestion

 Decreased wound healing

Consequences of a high central venous pressure

 Decreased venous return and stroke volume

 Acute kidney injury

 Hepatic congestion

 Decreased splanchnic microcirculatory flow

### The dangers of a normal saline

Despite differences in composition between normal saline (0.9% NaCl) and Lactated Ringer’s (LR), Normosol, Isolyte and Plasmalyte solutions, they are frequently considered physiologically equivalent. Whilst no body fluid has an electrolyte composition similar to that of normal saline (NS), this fluid is frequently referred to as ‘physiologic salt solution (PSS)’. However, 0.9% NaCl is more correctly known as ‘Unphysiologic Salt Solution’. Experimental and clinical data have clearly demonstrated that these fluids are not physiologically equivalent. Only LR, Normosol, Isolyte and Plasmalyte solutions are balanced salt solutions. Due to the calcium content of LR (and not Plasmalyte), it has been traditionally taught that LR should not be infused in the same venous line as blood (may activate clotting). However, this is not true [86].

### Renal failure

The increased chloride load delivered to the macula densa results in afferent arterial constriction [87]. In health volunteers, NS significantly reduces renal arterial flow and renal cortisol tissue perfusion as compared to Plasmalyte [88]. In a sequential cohort study Yunos and colleagues demonstrated that a chloride liberal fluid (NS) was associated with a much higher incidence of renal failure than critically patients resuscitated with a chloride restrictive fluid (LR and Plasmalyte) [32].

### Hyperchloremic metabolic acidosis and death

Numerous studies have demonstrated the development of a hyperchloremic metabolic acidosis in human volunteers and patients resuscitated with NS [89]–[92]. The additional loss (renal) of HCO_3_ in the setting of reduced buffering capacity only adds to the acid–base burden characteristic of hypoperfused states [90]. Furthermore, resuscitation with NS may produce a ‘dilutional acidosis’. In both experimental and clinical (pancreatitis) studies, hyperchloremic acidosis has been demonstrated to increase the release of inflammatory mediators [93],[94]. In a prospective cohort study, Boniatti *et al*. demonstrated that hyperchloremia was an independent predictor of death [95]. Similarly, in two large cohorts of patients undergoing non-cardiac surgery, hyperchloremia was an independently associated with increased morbidity and mortality [33],[34]. In these studies the risk of death increased with increasing chloride levels. Using a large multi-institutional database, Raghunathan *et al*. performed propensity score matching in patients with severe sepsis comparing resuscitation with balanced solutions versus NS [35]. In this study, resuscitation with a balanced solution was associated with a lower in-hospital mortality (19.6% versus 22.8%; relative risk (RR) 0.86; 95% CI, 0.78 to 0.94).

### Coagulopathy

Studies in surgical patients have demonstrated that as compared to LR, volume replacement with NS results in greater blood loss with a greater need for blood transfusion [91]. The cause of the coagulopathy is unclear, and is only partly explained by the difference in calcium between the two solutions.

### Lactate as a metabolic fuel

It is not an accidental quirk of nature that the body produces lactate during stress states. The proportion of lactate uptake by the myocardium and its use a metabolic fuel increases during exercise, β-adrenergic stimulation and shock [96]–[98]. During shock the heart undergoes a major shift in substrate utilization such that it oxidizes lactate for the majority of its energy needs [98]. Revelly and coworkers demonstrated that an infusion of sodium lactate increased cardiac performance in patients with both cardiogenic and septic shock [99]. Similarly, during increased demand on brain metabolism, lactate is increasingly utilized as an energy substrate [100]–[102]. LR may therefore have additional advantages in shocked patients with the lactate being oxidized and serving as a source of energy [103],[104].

## Conclusions

A liberal fluid resuscitation strategy, a CVP > than 8 mmHg and the use of 0.9% NaCl as the predominant resuscitation fluid are all associated with an increased risk of renal failure, respiratory failure, gastrointestinal dysfunction and death across a broad spectrum of clinical disorders. These three treatment strategies probably act synergistically to harm patients, forming the ‘Deadly Trio’.

## Abbreviations

AKI: acute kidney injury

ARDS: acute respiratory distress syndrome

ARISE: Australasian Resuscitation of Sepsis Evaluation trial

ATLS: Advanced Trauma Life Support

CVP: central venous pressure

CXR: chest radiograph

EGDT: Early Goal Directed Therapy

ESCAPE: Evaluation Study of Congestive Heart Failure and Pulmonary Artery Catheterization Effectiveness trial

EVLW: extra-vascular lung water

FACTT: Fluid and Catheter Treatment Trial

FEAST: The Fluid Expansion as Supportive Therapy study

GFR: glomerular filtration rate

LR: Lactated Ringer’s solution

MAP: mean arterial pressure

MCFP: mean circulatory filling pressure

NS: normal saline

ProCESS: Protocolized Care for Early Septic Shock trial

RR: relative risk

ScvO_2_: mixed venous oxygen saturation

VASST: Vasopressin in Septic Shock Trial

## Competing interest

The author has no real or perceived conflicts of interest or a financial interest in any of the products mentioned in this paper.

## Authors’ contribution

The author solely contributed to this paper and assumes full responsibility for the content of the paper.
